# Reduction in Dressing Change Burden in Patients With Epidermolysis Bullosa—Impact of Oleogel‐S10

**DOI:** 10.1111/1346-8138.17884

**Published:** 2025-08-01

**Authors:** Anna L. Bruckner, Dédée Murrell, Lara Wine Lee, Eli Sprecher, Dimitra Kiritsi, Laura Maher, Sandra Löwe, Maryanne Donovan, Johannes S. Kern, the EASE investigators

**Affiliations:** ^1^ Department of Dermatology University of Colorado School of Medicine Aurora Colorado USA; ^2^ Department of Dermatology, St. George Hospital University of new South Wales Sydney New South Wales Australia; ^3^ Department of Dermatology and Dermatologic Surgery Medical University of South Carolina Charleston South Carolina USA; ^4^ The Tel Aviv Sourasky Medical Center, Gray Faculty of Medicine and Health Sciences Tel Aviv University Tel Aviv Israel; ^5^ Department of Dermatology, Faculty of Medicine, Medical Center University of Freiburg Freiburg Germany; ^6^ First Department of Dermatology, Faculty of Medicine Aristotle University of Thessaloniki Thessaloniki Greece; ^7^ Amryt Research Limited Dublin Ireland; ^8^ Chiesi GRD Dublin Ireland; ^9^ Department of Dermatology, Alfred Hospital, School of Translational Medicine Monash University Melbourne Australia

## Abstract

Epidermolysis bullosa (EB) is a severe genetic disorder marked by skin fragility and blistering from minimal trauma. Management relies on frequent and painful dressing changes. The EASE study (NCT03068780), the largest to date in EB, previously demonstrated accelerated wound healing and reduced wound burden for Oleogel‐S10 (birch triterpenes) versus control gel. This post hoc analysis focused on dressing change frequency and related time and cost savings among patients with daily dressing changes at baseline (Oleogel‐S10 *n* = 47, control gel *n* = 53). By Day 90, 35.6% of Oleogel‐S10 patients required fewer daily changes versus 10.6% in the control group (*p* = 0.005). Weekly dressing changes reduced by 1.36 ± 0.24 with Oleogel‐S10 compared to 0.41 ± 0.23 for control (difference −0.95 ± 0.33; *p* = 0.005). This translated to almost three fewer dressing changes every 2 weeks for Oleogel‐S10 versus nearly one change for the control gel. The estimated time saved per week was 10.7 h with Oleogel‐S10 (6.4 h patient, 4.3 h caregiver) versus 4.0 h with control (2.4 h patient, 1.6 h caregiver). Estimated dressing costs reduced by 59%, from $63.4 k to $25.9 k per patient over 27 months. Oleogel‐S10 significantly reduced dressing frequency and time burden, with potential to ease the intensive demands of EB wound care.

## 1 Introduction

Epidermolysis bullosa (EB) is a rare genetic disease that causes severe mechanical fragility of the skin and other epithelial surfaces [1]. Major causes of death in EB include squamous cell carcinoma, septicemia, renal failure, cardiac failure, and amyloidosis [1, 2, 3, 4]. In its severe forms, EB is one of the most devastating diseases in humans [1].

Treatment of EB is focused on wound dressing [5]. Dressing changes are frequent [6] and can be as often as 1–2 times per day on uninfected wounds and 2–3 times per day on infected wounds. Given that approximately 32% of patients (across DEB, JEB and EB simplex) have wounds affecting > 30% of their body, the burden of repeated dressing changes is considerable [2]. Many patients require opioids and anxiolytics to relieve the pain associated with dressing changes [7]. Wound care is time‐consuming (~13% of patients spend > 4 h daily on this activity) [2] and costly [8]. These factors have a severe impact on private life [9] and finances [6]. Alongside the imperatives to heal wounds and prevent re‐injury and infection, EB patients and their caregivers have described decreasing time for dressing change as among one of the most important factors for EB therapies [2].

Oleogel‐S10 is a topical gel formulated with sunflower oil that contains 10% birch triterpenes [10]. In the Phase 3, double‐blind, randomized, vehicle‐controlled EASE trial (NCT03068780), Oleogel‐S10 demonstrated accelerated wound healing versus control gel in patients with EB [11] and was the first pharmaceutical agent approved for the treatment of EB. Here, we report analyses of daily dressing change frequency and estimated time and costs saved due to reduced daily dressing changes in patients treated with Oleogel‐S10.

## 2 Methods

The outcomes of EASE have been described previously [11, 12]. Patients with dystrophic or junctional EB with ≥ 1 partial‐thickness wound 10–50 cm^2^ present ≥ 21 days < 9 months were randomized by computer to receive topical Oleogel‐S10 or control gel along with standard‐of‐care dressings for 90 days (DBP) [11], with a subsequent switch of all patients to open‐label Oleogel‐S10 for a further 24 months (OLP) [12]. Study assessments were conducted at participating specialist centers. CONSORT information, protocol, and statistical analysis plan were published by Kern et al. [11]. EASE secured institutional review board approval from all participating centers and informed consent from all participants.

The frequency of dressing changes applied to all dressed wounds over time was captured at each visit. These data have been analyzed using analysis of covariance for all patients and the subset of patients with daily dressing changes at baseline (i.e., patients with the highest dressing burden). As the time needed for dressing changes was not captured in EASE, published evidence on the time required for whole‐body wound care [2] was used to calculate the estimated time saved for the subset of patients with daily dressing changes at baseline. An additional 66.7% of time spent by caregivers (defined by Bruckner et al.) [2] was added to the patient time to provide an overall estimate of time spent on daily dressing changes and time saved at Day 90.

Unit costs in America (US dollars) of a commonly used dressing (Mepitel) were used to calculate dressing costs. The cost of dressing changes over the DBP and OLP was calculated by adding the cost of dressings required to cover the body surface area percentage (BSAP) of all patients with daily dressing changes at baseline. The decreased cost at each visit after Day 0 (based on the reduced number of patients requiring daily dressing changes and the decreasing BSAP affected by EB wounds) was multiplied by the number of days between visits for the whole study period to provide the total cost—the same method was used to carry forward baseline data to estimate untreated costs.

## 3 Results

**FIGURE 1:**
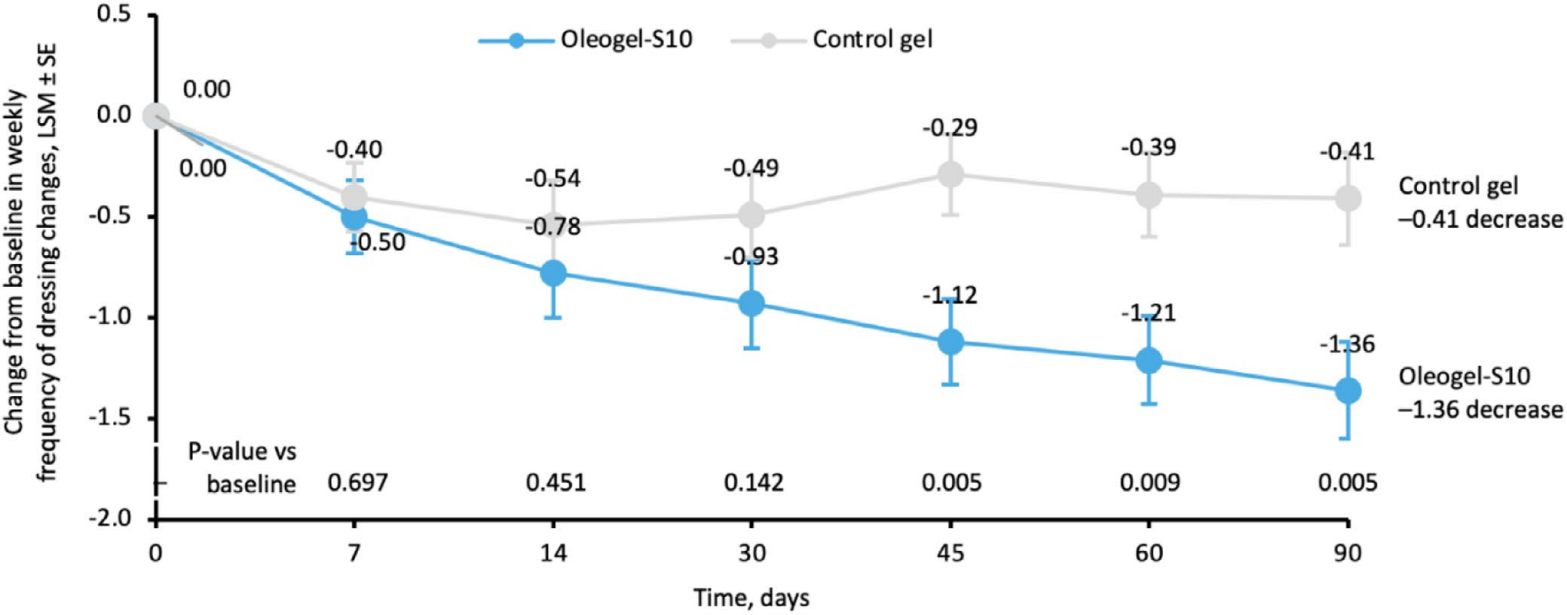
Changes from baseline in weekly frequency of dressing changes in the subgroup of patients with daily dressing changes at baseline in the EASE trial. Values represent LSM change in weekly frequency (±SE) of dressing changes in patients receiving daily dressing changes at baseline.

**FIGURE 2:**
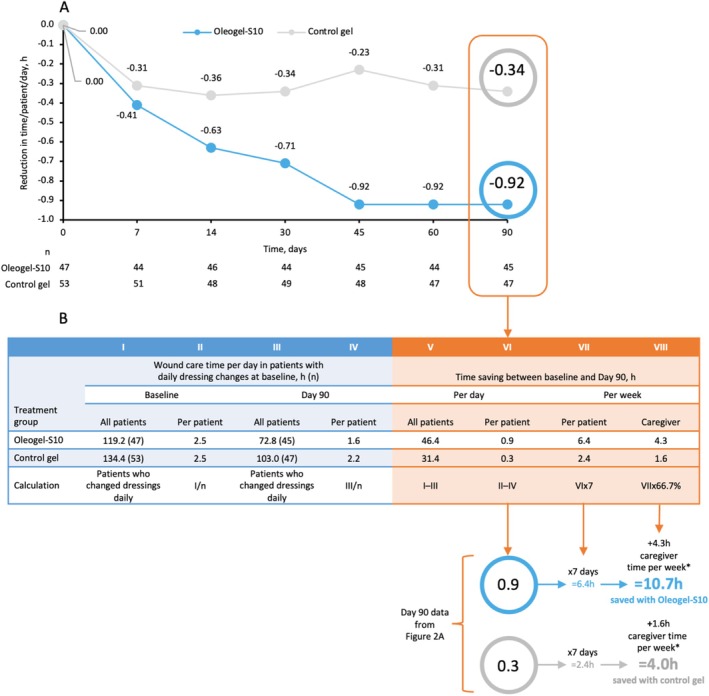
Reduction in time required for dressing changes by patient and visit (A) and ‘Estimation of total time saved from baseline by patient at Day 90 (B) in the subgroup of patients with daily dressing changes at baseline in the EASE trial. (A) Graphed values represent reduction in time spent per day per patient for patients receiving Oleogel‐S10 versus control gel. (B) Derivation of values using the difference in hours spent on daily changes between baseline and Day 90. Total hours spent are divided by the number of patients with daily changes to obtain the hours spent per day per patient. This value is multiplied by 7 days to obtain weekly values for time spent, and a calculation is applied using data from Bruckner et al. [2] that suggests that for every hour spent by patients, an additional 66.7% is spent by caregivers. *66.7% of patients require assistance with dressing changes Bruckner et al. [2] LSM, least squares mean; SE, standard error of the mean.

Oleogel‐S10 demonstrated accelerated wound healing in patients with EB. A selection of target wound images demonstrating wound healing over time for patients treated with Oleogel‐S10 is shown in Figure S1 [11]. Among patients with once‐daily dressing changes at baseline (Figure S2), a higher percentage of Oleogel‐S10 patients no longer required daily dressing changes versus control gel at Day 90 (35.6% vs. 10.6%; *p* = 0.005; Figure 1; Table S1). The change at Day 90 in the number of dressing changes required each week in this subset was −1.36 changes for Oleogel‐S10 versus −0.41 changes for control gel (*p* = 0.005; Figure 1). This equates to almost three fewer dressing changes every 2 weeks for Oleogel‐S10 versus only one fewer for control gel. At baseline, 43% of patients in the Oleogel‐S10 group and 46% of patients in the control gel group were undergoing daily dressing changes, and using the Bruckner category analysis, this was estimated to equate to 119.2 h and 134.4 h spent on dressing changes per treatment group, respectively (Figure 2). At Day 90, these values had reduced to 72.8 h and 103.0 h per treatment group, respectively, resulting in time saved per patient of 6.4 h per week for Oleogel‐S10 versus 2.4 h per week for control gel (Figure 2). Adjusting for caregiver time using the Bruckner data, the total time saved per week for each Oleogel‐S10 patient was 10.7 h versus 4.0 h for each control gel patient, respectively (Figure 2).

**FIGURE 3:**
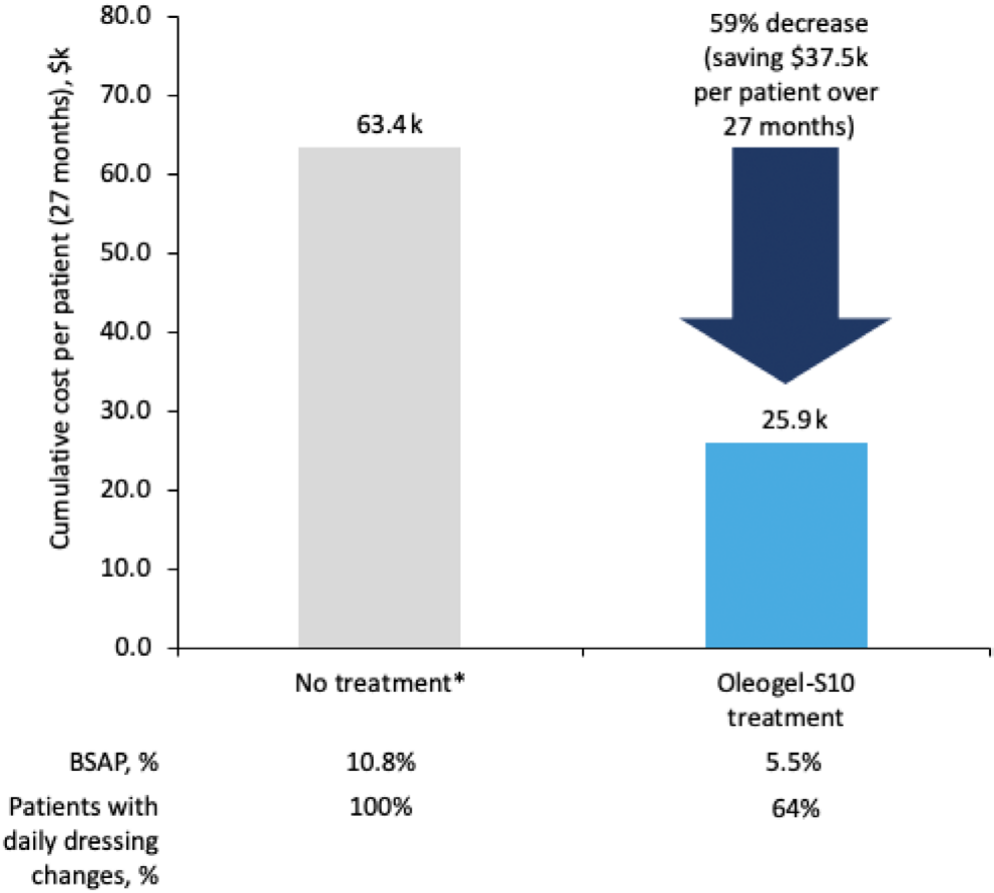
Costs saved on dressing changes: Derived from EASE data over 27 months. Data derived from the subgroup of patients requiring daily dressing changes at baseline. *The total cost of dressing changes for untreated patients calculated by multiplying by the cost of dressings required to cover the BSAP of all patients with daily dressing changes at Baseline for the treatment duration for DBP (91 days) and OLP (2 years). Unit costs in America (US dollars) for Mepitel were used to estimate dressing costs. (Mepitel was one of a small number of dressings that were recommended in EASE to reduce the diversity of wound dressings as much as possible; however, not all patients in EASE used Mepitel dressings). BSAP, body surface area percentage; DBP; double‐blind phase; k thousand; OLP, open‐label phase.

Accounting for decreased dressing change frequency and reduction in mean BSAP from 10.8% to 5.5% (for the sub‐group of patients with daily dressing changes at baseline), the estimated cost of dressings was reduced by ~60%, from $63.4 k per patient over 27 months if untreated to $25.9 k with Oleogel‐S10 treatment (Figure 3). This represents annualized costs saved of $16.7 k per patient. In the EASE cohort, this could have translated to a $3.3 million saving throughout the study for patients who required daily dressing changes at baseline.

## 4 Discussion

The analysis of EASE described here shows that Oleogel‐S10 reduced the requirement for daily dressing changes and corresponding time spent on this painful procedure. EB places an enormous burden upon patients and families, and reducing the time spent on dressing changes is an important objective for patients and caregivers [2]. Additionally, dressing changes can be extremely painful, and steps that can be taken to moderate them are welcomed by patients with EB [6]. A reduction in procedural pain (pain associated with dressing changes) has previously been reported in patients treated with Oleogel‐S10 (Day 14: Oleogel‐S10, −1.4 versus −0.8 control gel; *p* = 0.02; decrease in the Wong‐Baker FACES pain scale in patients ≥ 4 years old) [11]. Overall, these data indicate that Oleogel‐S10 reduces the burden of dressing changes, which potentially positively impacts the severity of pain experienced by patients. These findings are supported by previous real‐world data that reports reduced frequency of dressing changes and wound burden over time for patients receiving Oleogel‐S10 [13]. Importantly, despite the reduced frequency of dressing changes, there were no associated increases in infections for Oleogel‐S10 versus control gel, and infections that did occur were of a lower frequency and lesser severity for Oleogel‐S10 versus control gel [11].

Specialized dressings and complex dressing regimes, particularly for severe patients, give rise to high financial pressure on both families and healthcare systems. Mean monthly costs for patients and caregivers can be very high—the Woundcare for Epidermolysis Bullosa Project has estimated monthly costs as high as £35 865/month [14], and the PEBLES study has placed annual costs as high as £542 543 per year [15]. Annual costs of EB care are routinely underestimated by healthcare systems [6]. Patients are frequently required to fill any gaps in provision, even re‐using dressings, which carries an infection risk [6]. Decreasing the frequency of dressing changes in EASE, together with BSAP reductions, led to considerably reduced dressing costs, potentially relieving some of the financial burden experienced by EB patients.

Limitations of this analysis include the assumption of a static position on BSAP and dressing change frequency with no active treatment, and reliance on published evidence to calculate time spent on dressing changes in EASE.

In conclusion, the data presented here show that Oleogel‐S10 has the potential to reduce the burden of dressing changes, a painful, distressing, and costly procedure that is a constant challenge for those living with EB.

## Conflicts of Interest

A.L.B. has received grants and/or fees for consultancy in the last 12 months from Amryt/Chiesi, Castle Creek, Krystal Biotech, RHEACELL GmbH, and Twi Biotechnology. D.M. has received grants and/or fees for consultancy in the last 12 months from Amryt/Chiesi, Amicus, and is a co‐patent owner for topical sirolimus for EBS. L.W.L. has received grants and/or fees for consultancy in the last 12 months from Amryt/Chiesi, Krystal Biotech, Castle Creek, and Twi Biotechnology. E.S. has received grants for consultancy in the last 12 months from Amryt/Chiesi, Kamari Pharma Ltd., BiomX, and as CMO of Sol–Gel Technologies Ltd. D.K. has received grants/research support from RHEACELL GmbH, honoraria or consultation fees from Amryt/Chiesi, RHEACELL GmbH, and Fibrx Derm Inc., and research funding from DEBRA International, EB Research Partnership, Fritz‐Thyssen Foundation, and German Research Foundation. L.M. was an employee of Amryt Research Ltd. at the time of this analysis. S.L. is a contractor for Chiesi GRD via Löwe Medical Consulting & Support, Munich, Germany. M.D. is an employee of Chiesi GRD. J.S.K. reports grants from Amryt during the conduct of the study, and other grants from AbbVie, Amicus, Amgen, Amryt/Chiesi, Arcutis, Argenix, Arena, Astra‐Zeneca, Boehringer Ingelheim, BMS, Cutanea, CSL, DFG/Fresenius, Dompé, Eli Lilly, Evolo, Exopharm, Galderma, Genentech, Incyte, InflaRx, Kiniksa, Merck KGaA, Mitsubishi, Novartis, Pfizer, Principia, Regeneron, Sanofi, Shire, Scioderm, Takeda, and UCB outside the submitted work.

## Supporting information


**Data S1:** jde17884‐sup‐0001‐Supplemental Material.pdf.

## Data Availability

The data that support the findings of this study are available on request from the corresponding author. The data are not publicly available due to privacy or ethical restrictions.

## References

[1] J. D. Fine , “Inherited Epidermolysis Bullosa,” Orphanet Journal of Rare Diseases 5 (2010): 12, 10.1186/1750-1172-5-12. 20507631 PMC2892432

[2] A. L. Bruckner , M. Losow , J. Wisk , et al., “The Challenges of Living With and Managing Epidermolysis Bullosa: Insights From Patients and Caregivers,” Orphanet Journal of Rare Diseases 15, no. 1 (2020): 1, 10.1186/s13023-019-1279-y. 31900176 PMC6942340

[3] C. Has , J. W. Bauer , C. Bodemer , et al., “Consensus Reclassification of Inherited Epidermolysis Bullosa and Other Disorders With Skin Fragility,” British Journal of Dermatology 183, no. 4 (2020): 614–627, 10.1111/bjd.18921. 32017015

[4] J. D. Fine , “Epidemiology of Inherited Epidermolysis Bullosa Based on Incidence and Prevalence Estimates From the National Epidermolysis Bullosa Registry,” JAMA Dermatology 152, no. 11 (2016): 1231–1238, 10.1001/jamadermatol.2016.2473. 27463098

[5] E. Pope , I. Lara‐Corrales , J. Mellerio , et al., “A Consensus Approach to Wound Care in Epidermolysis Bullosa,” Journal of the American Academy of Dermatology 67, no. 5 (2012): 904–917, 10.1016/j.jaad.2012.01.016. 22387035 PMC3655403

[6] P. Grocott , R. Blackwell , H. Weir , and E. Pillay , “Living in Dressings and Bandages: Findings From Workshops With People With Epidermolysis Bullosa,” International Wound Journal 10, no. 3 (2013): 274–284, 10.1111/j.1742-481X.2012.00973.x. 22487531 PMC7950808

[7] K. R. Goldschneider , J. Good , E. Harrop , et al., “Pain Care for Patients With Epidermolysis Bullosa: Best Care Practice Guidelines,” BMC Medicine 12 (2014): 178, 10.1186/s12916-014-0178-2. 25603875 PMC4190576

[8] J. A. Feinstein , P. Jambal , K. Peoples , et al., “Assessment of the Timing of Milestone Clinical Events in Patients With Epidermolysis Bullosa From North America,” JAMA Dermatology 155, no. 2 (2019): 196–203, 10.1001/jamadermatol.2018.4673. 30586139 PMC6439540

[9] J. Y. Tang , M. P. Marinkovich , E. Lucas , et al., “A Systematic Literature Review of the Disease Burden in Patients With Recessive Dystrophic Epidermolysis Bullosa,” Orphanet Journal of Rare Diseases 16, no. 1 (2021): 175, 10.1186/s13023-021-01811-7. 33849616 PMC8045359

[10] M. Laszczyk , S. Jager , B. Simon‐Haarhaus , A. Scheffler , and C. M. Schempp , “Physical, Chemical and Pharmacological Characterization of a New Oleogel‐Forming Triterpene Extract From the Outer Bark of Birch (Betulae Cortex),” Planta Medica 72, no. 15 (2006): 1389–1395, 10.1055/s-2006-951723. 17091432

[11] J. S. Kern , E. Sprecher , M. F. Fernandez , et al., “Efficacy and Safety of Oleogel‐S10 (Birch Triterpenes) for Epidermolysis Bullosa: Results From the Phase III Randomized Double‐Blind Phase of the EASE Study,” British Journal of Dermatology 188, no. 1 (2023): 12–21, 10.1093/bjd/ljac001. 36689495

[12] D. F. Murrell , C. Bodemer , A. L. Bruckner , et al., “Long‐Term Safety and Efficacy of Oleogel‐S10 (Birch Bark Extract) in Epidermolysis Bullosa: 24‐Month Results From the Phase III EASE Study,” British Journal of Dermatology 192 (2025): 1007–1017, 10.1093/bjd/ljaf022. 39821055

[13] M. Torres Pradilla , E. Alvarez , M. Novoa , I. Lozano , and M. Trujillo , “Oleogel‐S10 in Dystrophic Epidermolysis Bullosa: A Case Series Evaluating the Impact on Wound Burden Over Two Years,” Advances in Therapy 41, no. 2 (2024): 867–877, 10.1007/s12325-023-02749-x. 38170434 PMC10838820

[14] P. Grocott , T. Graham , R. Blackwell , et al., “Individualising Wound Care Research: The Woundcare for Epidermolysis Bullosa Project,” Wounds 9, no. 3 (2013): 23–32.

[15] J. Mellerio , PEBLES: Natural History of EB (DEBRA International, 2016).

