# Supplementary material for: Reduction in Dressing Change Burden in Patients With Epidermolysis Bullosa—Impact of Oleogel‐S10
Source: J Dermatol. 2025 Aug 1;52(9):1447–51. doi: 10.1111/1346-8138.17884 (PMC12411827; doi:10.1111/1346-8138.17884)
Supplement: Supplementary file 1 — Data S1: jde17884‐sup‐0001‐Supplemental Material.pdf. [file JDE-52-1447-s001.pdf]

Supplemental Figure 1. Wound healing over time for patients treated with Oleogel-S10 in the EASE trial

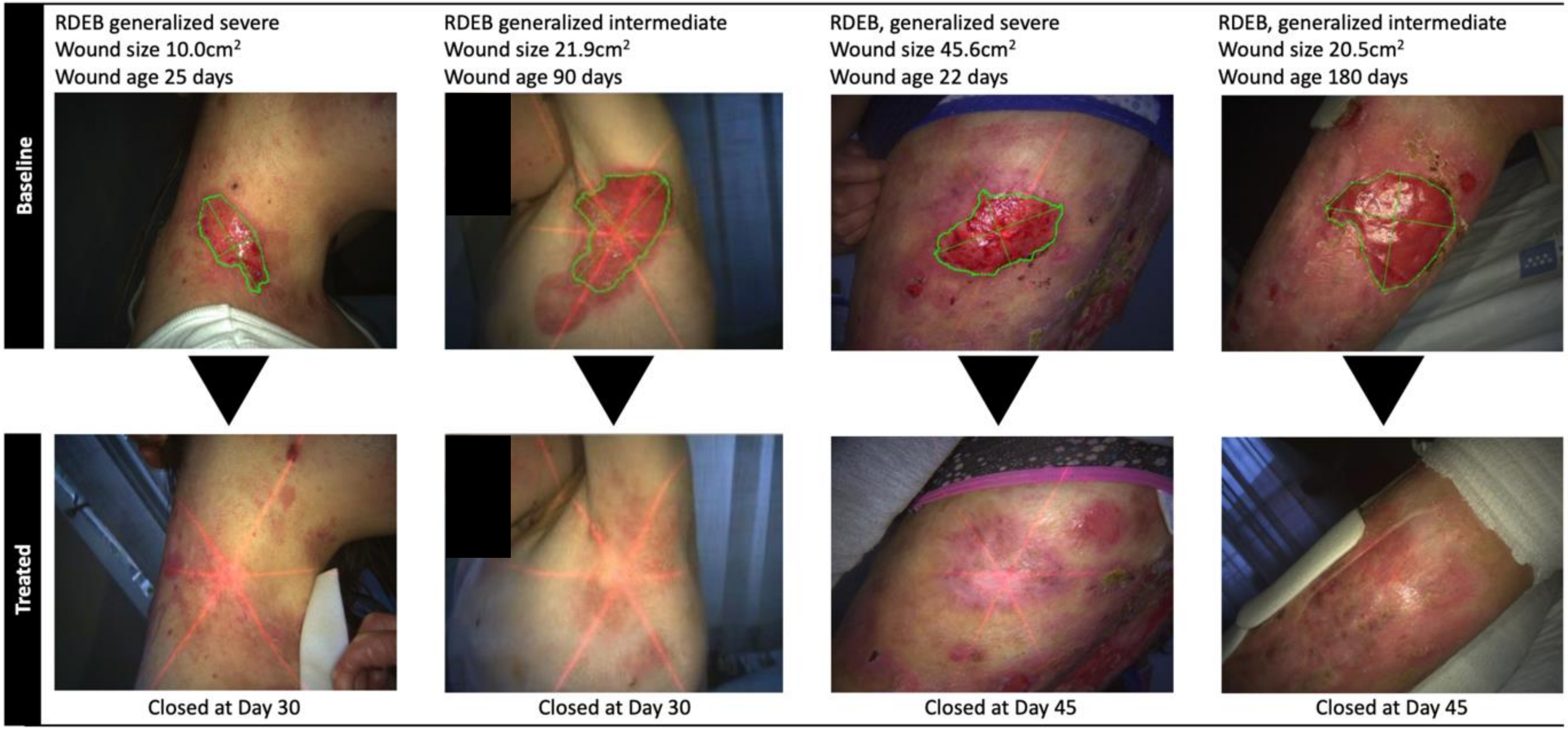

Figure reproduced with permission from Kern JS, Sprecher E, Fernandez MF, et al. Efficacy and safety of Oleogel-S10 (birch triterpenes) for epidermolysis bullosa: results from the phase III randomized double-blind phase of the EASE study. Br J Dermatol. Jan 23 2023;188(1):12-21. doi:10.1093/bjd/ljac001

Images are representative of patients receiving Oleogel-S10 in the EASE trial.

RDEB, recessive dystrophic epidermolysis bullosa

Supplemental Figure 2. CONSORT flow diagram

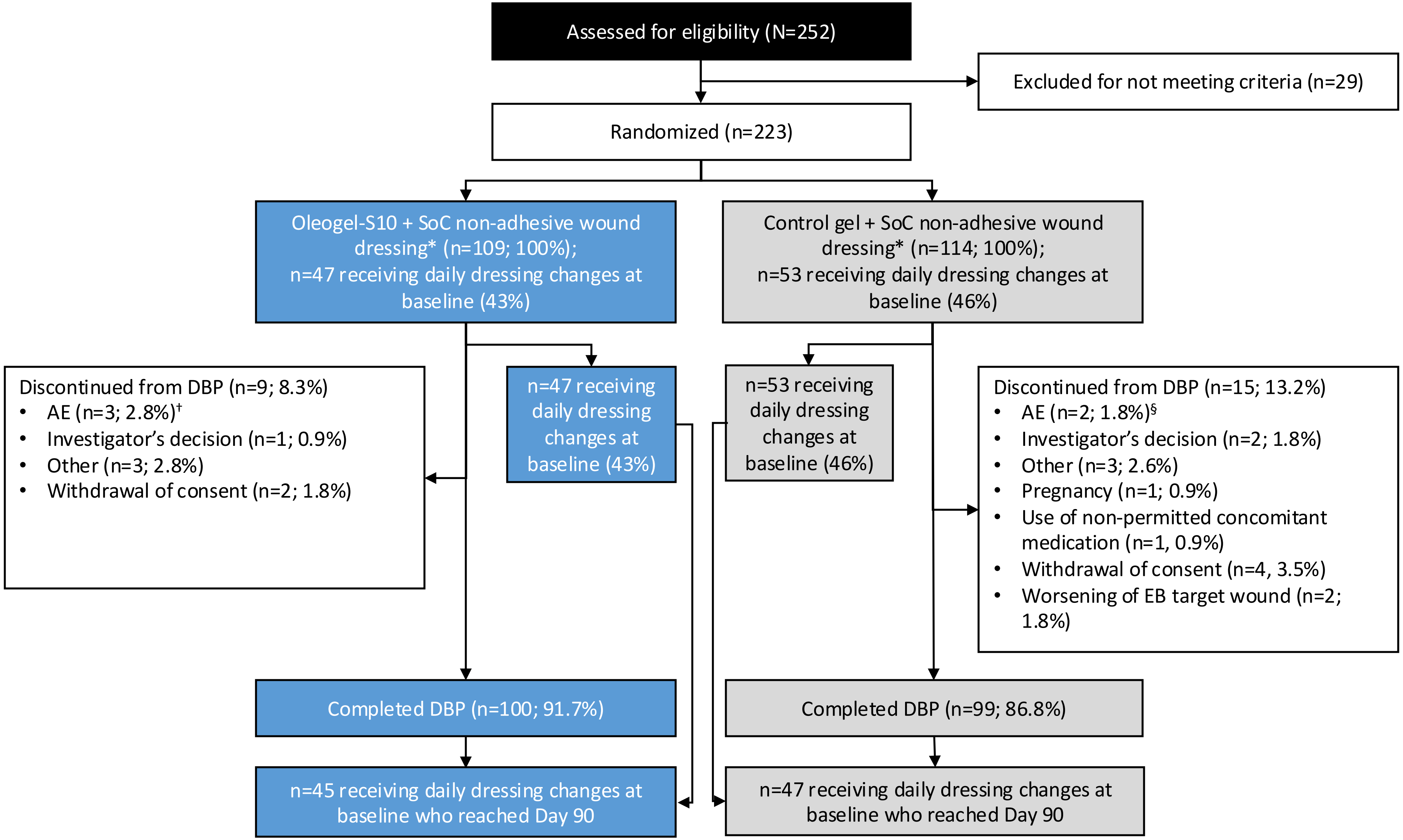

*Standard of care non-adhesive wound dressings were defined as modern non-adhesive wound dressings. To reduce the diversity of wound dressings as much as possible, a small number of products, such as Mepitel® or PolyMem® (or equivalents) was recommended and any changes in dressing type during the study were documented.

†1 patient with procedural pain, 1 patient with squamous cell carcinoma, 1 patient with wound hemorrhage

§1 patient with allergic rash, 1 patient with wound complication, 1 patient with increase in wound size compared to baseline.

AE, adverse event; DBP, double-blind phase; EB, epidermolysis bullosa; SoC, standard of care

Supplemental Table 1. Calculation of time saved for patients with daily dressing changes at baseline in the EASE trial

		Time Required (Bruckner et al.)*			Oleogel-S10			Control Gel			
					47		Time required (h)	53			Time required (h)
**Overall**	n	1h	3h	5h	Daily	<Daily		n	Daily	<Daily	
**Baseline**					47	0			53	0	
	**47**				100%			**53**	100%		
RDEB	33	37.0%	26.0%	37.0%	33	0	99.0	37	37	0	111.0
DDEB	6	79.0%	21.0%		6	0	8.5	7	7	0	9.9
JEB	7	75.0%	25.0%		7	0	10.5	9	9	0	13.5
EBS	1	95.2%	0.0%	4.80%	1	0	1.2	0	0	0	0.0
Total Time Required (Subgroup)							**119.2**				**134.4**
Time Required (Subject)							**2.54**				**2.54**
**D7**	44				37	7		51	45	6	
	**44**				84.1%	15.9%		**51**	88.2%	11.8%	
RDEB	31				26	5	78.0	35	31	4	93.0
DDEB	6	See baseline values			6	0	8.5	7	6	1	8.5
JEB	6				4	2	6.0	9	8	1	12.0
EBS	1				1	0	1.2	0	0	0	0.0
Total Time Required (Subgroup)							**93.7**				**113.5**
Time Required (Subject)							**2.13**				**2.23**
**D14**	46				35	11		48	42	6	
	**46**				76.1%	23.9%		**48**	87.5%	12.5%	
RDEB	32				24	8	72.0	32	28	4	84.0
DDEB	6	See baseline values			5	1	7.1	7	5	2	7.1
JEB	7				5	2	7.5	9	9	0	13.5
EBS	1				1	0	1.2	0	0	0	0.0
Total Time Required (Subgroup)							**87.8**				**104.6**
Time Required (Subject)							**1.91**				**2.18**
**D30**	44				32	12		49	42	7	
	**44**				72.7%	27.3%		**49**	85.7%	14.3%	
RDEB	30				22	8	66.0	33	30	3	90.0
DDEB	6	See baseline values			5	1	7.1	7	4	3	5.7
JEB	7				4	3	6.0	9	8	1	12.0
EBS	1				1	0	1.2	0	0	0	0.0
Total Time Required (Subgroup)							**80.3**				**107.7**
Time Required (Subject)							**1.82**				**2.20**
**D45**	45				30	15		48	43	5	
	**45**				66.7%	33.3%		**48**	89.6%	10.4%	
RDEB	31				19	12	57.0	33	31	2	93.0
DDEB	6	See baseline values			5	1	7.1	6	4	2	5.7
JEB	7				5	2	7.5	9	8	1	12.0
EBS	1				1	0	1.2	0	0	0	0.0
Total Time Required (Subgroup)							**72.8**				**110.7**
Time Required (Subject)							**1.62**				**2.31**
**D60**	44				29	15		47	41	6	
	**44**				65.9%	34.1%		**47**	87.2%	12.8%	
RDEB	30				19	11	57.0	33	29	4	87.0
DDEB	6	See baseline values			5	1	7.1	6	5	1	7.1
JEB	7				4	3	6.0	8	7	1	10.5
EBS	1				1	0	1.2	0	0	0	0.0
Total Time Required (Subgroup)							**71.3**				**104.6**
Time Required (Subject)							**1.62**				**2.23**
**D90**	45				29	16		47	42	5	
	**45**				64.4%	35.6%		**47**	89.4%	10.6%	
RDEB	31				20	11	60.0	31	27	4	81.0
DDEB	6	See baseline values			5	1	7.1	7	6	1	8.5
JEB	7				3	4	4.5	9	9	0	13.5
EBS	1				1	0	1.2	0	0	0	0.0
Total Time Required (Subgroup)							**72.8**				**103.0**
Time Required (Subject)							**1.62**				**2.19**
**Change from Baseline**											
Total Time Saved (Subgroup) per Day (h)							46.4				31.4
Time Saved (Subject) per Day (h)							0.92				0.34
**Time Saved (Subject) per Week (h)**							6.43				2.4
**Time Saved (Assistant Caregiver) per Week (h)**							4.29				1.6
**Total Time Saved (Subject & Caregiver) per Week (h)**							**10.72**				**4.0**
**Total Time Saved (Subject & Caregiver) per Month (h)**							46.0				17.2
**Total Time Saved (Subject & Caregiver) per Month (days)**							1.9				0.7

*As the time needed for dressing changes was not captured in EASE, published evidence on the time required for whole-body wound care (Bruckner AL, et al. Orphanet J Rare Dis 2020;15:1) was used to calculate the estimated time saved for the subset of patients with daily dressing changes at baseline. An additional 66.7% of time spent by caregivers (defined by Bruckner et al) was added to the patient time to provide an overall estimate of time spent on daily dressing changes and time saved at Day 90.
